# Angiogenic and Regenerative Potential of Plasma-Based and Plasma-Free Platelet Concentrates Obtained via Different Fractionation and Activation Methods

**DOI:** 10.3390/bioengineering13070821

**Published:** 2026-07-16

**Authors:** Irina Alekseevna Nedorubova, Viktoriia Pavlovna Basina, Anastasiia Yurevna Meglei, Maria Gennadevna Sapelnikova, Anatoly Alekseevich Kulakov, Dmitry Vadimovich Goldshtein, Tatiana Borisovna Bukharova

**Affiliations:** 1 Research Centre for Medical Genetics, 115478 Moscow, Russia; vika.basina12@gmail.com (V.P.B.); an.megley95@yandex.ru (A.Y.M.); krasnova.m.g.0605@gmail.com (M.G.S.); dvgoldshtein@gmail.com (D.V.G.); bukharova-rmt@yandex.ru (T.B.B.); 2 Central Research Institute of Dental and Maxillofacial Surgery, 119021 Moscow, Russia

**Keywords:** PRP, platelet concentrates, plasma-free platelet concentrate, tissue engineering, angiogenesis, cell migration, regenerative dentistry

## Abstract

Background: Platelet concentrates are widely used in regenerative dentistry and maxillofacial surgery; however, the lack of protocol standardization and selection criteria results in contradictory clinical outcomes. This study aimed to perform a comparative analysis of the biological activity of various platelet concentrates obtained via different fractionation and activation procedures under uniform in vitro experimental conditions. Methods: Rat adipose-derived stem cells (ADSCs) and human EA.hy926 endothelial cells were used to evaluate four platelet concentrate types via tube formation, wound healing (scratch), and cell proliferation assays. Results: Platelet concentrates obtained by the single-centrifugation protocol (L-PRP-1) exhibited maximal retained platelet potential, corresponding to a peak 5-fold increase in cell migration. Chemical activation of plasma-based concentrates (L-PRP, P-PRP) with calcium/thrombin was critically required to trigger angiogenesis and accelerate endothelial chemotaxis. Conversely, plasma-free platelet concentrate (PFPC) exhibited a unique capacity for spontaneous activation and clot formation without exogenous inducers, triggering rapid angiogenesis and sustaining ADSC proliferation. Conclusions: Within the framework of our in vitro model, activated plasma-based forms are biologically justified for accelerated soft tissue healing and socket preservation, whereas the complete removal of plasma proteins suggests the potential utility of PFPC as a biomimetic matrix-carrier for maxillofacial tissue engineering.

## 1. Introduction

Platelet concentrates are widely used in regenerative medicine, dentistry, traumatology, and tissue engineering as a source of biologically active molecules that stimulate wound healing. The most common forms of platelet concentrates are platelet-rich plasma (PRP) and its modifications, which differ in leukocyte content and the presence of plasma proteins [1]. Platelet concentrates occupy a special place among orthobiologic products because they not only contain regeneration-enhancing growth factors and cytokines within their granules but can also serve as biomimetic scaffolds for delivering cells and genetic constructs into tissues [2,3,4]. Furthermore, PRP is derived from the patient’s own blood, which reduces the likelihood of immune rejection and ensures high biocompatibility [5].

The composition, properties, and, consequently, the biological effects of platelet concentrates depend on the preparation method. Various isolation protocols exist, differing in the number of centrifugation steps, speed, duration, and final product composition [6]. It is well established that platelet activation triggers the release of various bioactive factors from their granules, which exert pro-angiogenic, chemotactic, and mitogenic effects; this directly promotes angiogenesis, cell migration, and proliferation—the key processes of tissue regeneration [7,8]. However, the question of the most effective methods for activating platelet concentrates, particularly the necessity of pre-treatment with calcium and thrombin solutions, remains a matter of debate.

The application of PRP has become an integral part of regenerative protocols in modern dental implantology [9]. Local administration of autologous concentrates is critically important for achieving successful guided bone regeneration in alveolar ridge defects, for performing sinus lift procedures, and for stimulating tissue recovery in the treatment of severe periodontitis. Enriching the surgical site with growth factors not only accelerates bone graft maturation and soft tissue healing but also significantly enhances osseointegration, improving the quality of the bone-to-implant contact [10].

Despite the large number of studies on PRP, data on its clinical efficacy remain contradictory, primarily due to the lack of standardization in centrifugation protocols and criteria for selecting specific concentrate types for targeted clinical applications in regenerative dentistry. In this regard, a comparative analysis of the functional activity of various platelet concentrate types under uniform experimental conditions, using a unified assessment of their biological activity, represents a critical research direction.

The aim of this study was to perform a comparative analysis of the angiogenic, proliferative, and migratory activities of various platelet concentrates obtained via different fractionation and activation procedures under uniform in vitro experimental conditions.

## 2. Materials and Methods

### 2.1. Cell Cultures

Rat adipose-derived stem cells (ADSCs), previously isolated and characterized [11], and the immortalized human endothelial cell line EA.hy926 (CRL-2922; ATCC, Manassas, VA, USA) were used in this study. EA.hy926 is generated by hybridizing primary HUVECs (human umbilical vein endothelial cells) with the A549 lung carcinoma cell line. As a result, EA.hy926 cells retain the core functional characteristics of primary endothelium and are widely recognized as a reliable and well-validated alternative to primary cultures for angiogenesis research. However, it is not a primary endothelial cell, which should be taken into account when interpreting the results. Cells were cultured in DMEM/F12 medium (PanEco, Moscow, Russia) supplemented with 10% fetal bovine serum (BioWest, Nuaillé, France), 0.584 mg/mL L-glutamine (PanEco, Moscow, Russia), 5000 U/mL penicillin, and 5000 μg/mL streptomycin (PanEco, Moscow, Russia) at 37 °C in a humidified atmosphere containing 5% CO_2_. The culture medium was replaced every three days.

### 2.2. Platelet Concentrates

To prepare platelet concentrates—specifically PRP and plasma-free platelet concentrate (PFPC)—blood was collected from Wistar rats via cardiac puncture, gathered into tubes containing sodium citrate, and then centrifuged under several conditions (Table 1). Blood cell quantification was performed using a Hemalight 1280 hematology analyzer (Dixion, Moscow, Russia). A portion of the platelet concentrates was activated prior to being added to the cells. For platelet activation, 20 μL of a thrombin solution (100 NIH; PZ Cormay, Warsaw, Poland) in a 10% calcium chloride solution (NPO Microgen, Moscow, Russia) was added to 80 μL of the platelet concentrates.

### 2.3. Tube Formation Assay

To evaluate the angiogenic potential of the platelet concentrates, the EA.hy926 cell line was used. The cells were seeded into 24-well plates (SPL Life Sciences, Pocheon-si, Republic of Korea), and after 24 h, the platelet concentrates were added. Tubular network formation was assessed over a 48-h period via phase-contrast microscopy using a Lionheart FX microscope (BioTek Instruments Inc., Santa-Clara, CA, USA).

### 2.4. Cell Migration Assay

The influence of different types of platelet concentrates on the migration of EA.hy926 endothelial cells was investigated using a wound healing (scratch) assay. EA.hy926 cells were seeded into 24-well plates, cultured to full confluence, and a monolayer wound was created. Detached cells were washed away, growth medium was added, and various types of platelet concentrates were introduced. Endothelial cell migration was monitored over a 48-h period using a Lionheart FX microscope. The confluence of the monolayer wound area was evaluated using ImageJ software 1.52a (National Institutes of Health, Bethesda, MD, USA).

### 2.5. ADSC Proliferation Assay

The influence of different types of platelet concentrates on cell proliferative activity was investigated using ADSCs pre-stained with PKH26 (Sigma-Aldrich, St. Louis, MO, USA) according to the manufacturer’s instructions, via fluorescence microscopy using a Lionheart FX microscope. To quantify ADSC proliferation, changes in cell number per well were evaluated after 1 and 2 days compared to the baseline count. For this purpose, the cells were detached from the substrate using a Trypsin-Versene solution (PanEco, Moscow, Russia) and analyzed using an automated cell counter (Bio-Rad, Hercules, CA, USA).

### 2.6. Statistical Analysis

At least 4 biological replicates were used in each experiment, with each biological replicate consisting of 3 technical replicates. Data visualization and statistical analysis were performed using SigmaPlot v14.0 (Systat Software Inc., Palo Alto, CA, USA). The normality of data distribution was assessed using the Kolmogorov–Smirnov test with the Lilliefors correction. Homogeneity of variances among experimental groups was evaluated using Levene’s test. All datasets met the assumptions of normality and homogeneity of variances, confirming the appropriateness of parametric statistical methods. Intergroup differences were statistically analyzed using a one-way ANOVA followed by the Holm–Sidak post hoc test. Data are presented as mean ± standard deviation (SD).

## 3. Results

### 3.1. Characterization of Platelet Concentrates

In this study, L-PRP, P-PRP, and PFPC were obtained. All preparation protocols resulted in an increased platelet concentration (Table 2) and a decreased leukocyte concentration (Table 3) compared to whole blood. Following a single centrifugation step, the platelet concentration increased approximately 3-fold, whereas a two-step centrifugation protocol resulted in an approximate 6- to 8-fold enrichment.

### 3.2. Effects of Platelet Concentrates on Angiogenesis

**Figure 1:**
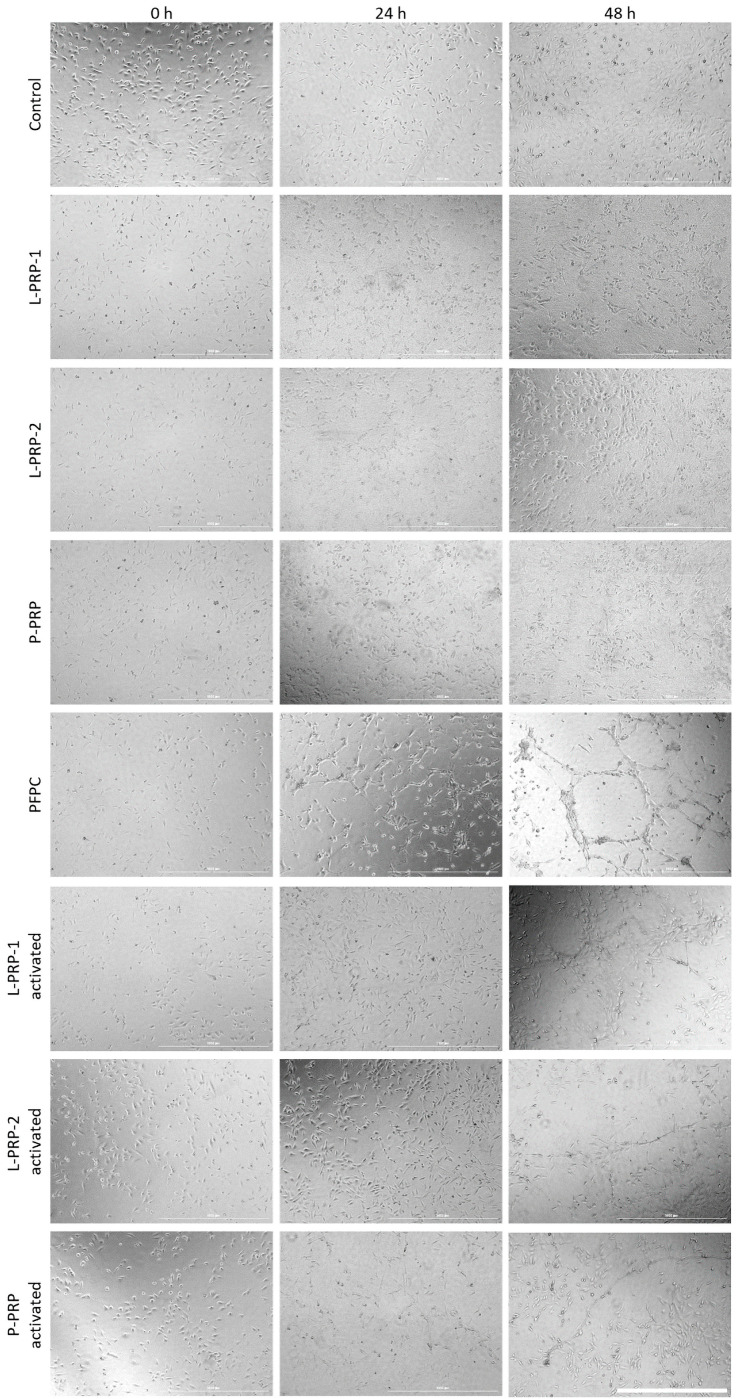
Effects of different types of platelet concentrates on tube formation by EA.hy926 endothelial cells. Phase-contrast microscopy images. Scale bar: 1000 µm.

The effects of platelet concentrates obtained by different protocols on angiogenesis were evaluated using the EA.hy926 endothelial cell line. Tube-like structure formation was observed after 48 h only in groups treated with L-PRP-1, L-PRP-2, or P-PRP that had been pre-activated by the addition of thrombin in calcium chloride solution (Figure 1). Pre-activation of plasma-based platelet concentrates significantly promoted endothelial tube network formation, whereas no such effect was observed in non-activated groups.

In contrast, when EA.hy926 cells were incubated with PFPC, the initiation of tube network formation was observed as early as 24 h and persisted through 48 h (Figure 1). Furthermore, the addition of PFPC to the cells induced platelet activation and fibrin clot formation in the wells, which was not observed with the other non-activated plasma-based PRP types.

### 3.3. Effects of Platelet Concentrates on Cell Migration in a Wound Healing Assay

**Figure 2:**
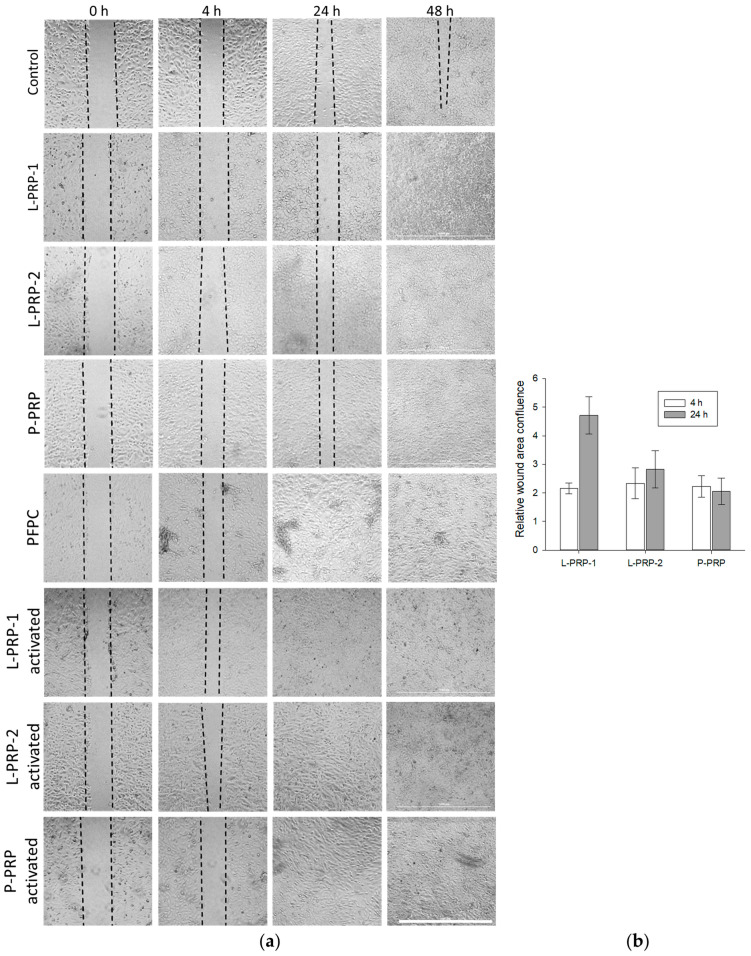

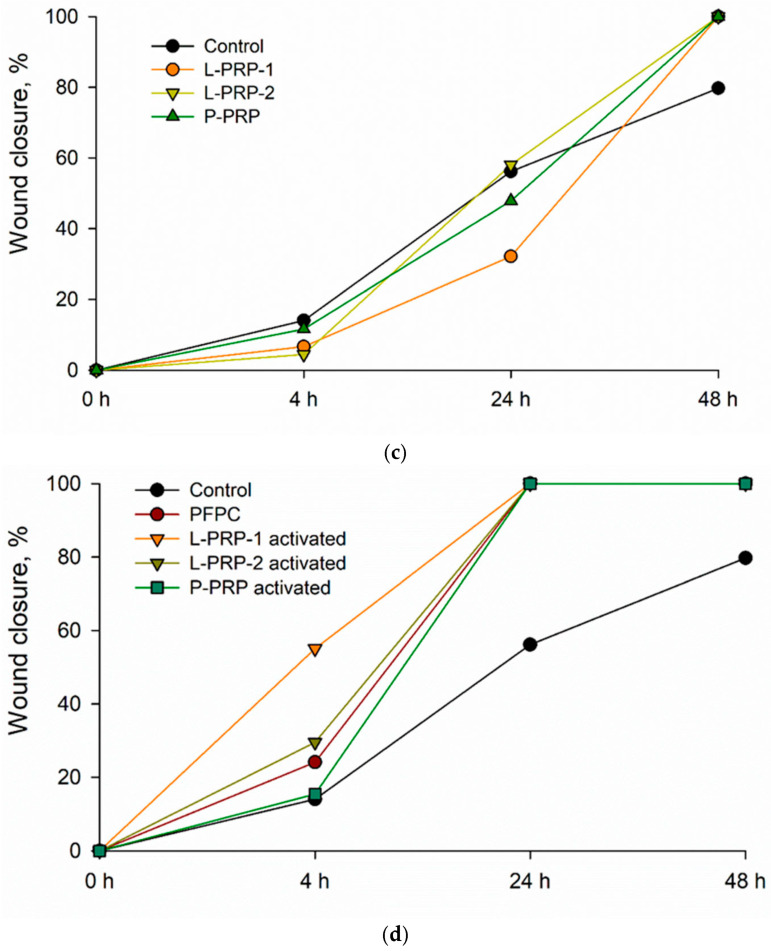
Effects of different types of platelet concentrates on the wound healing capacity of EA.hy926 endothelial cells in a scratch assay: (**a**) Representative phase-contrast microscopy images of the cell monolayer. The dashed line represents the edges of the monolayer wound. Scale bar: 1000 µm. (**b**) Quantitative analysis of wound confluence relative to the corresponding non-activated platelet concentrate types. (**c**) Kinetic curves showing the percentage of wound closure after the addition of non-activated platelet concentrates. (**d**) Kinetic curves showing the percentage of wound closure after the addition of activated platelet concentrates.

The effects of different types of platelet concentrates on the migratory activity of EA.hy926 endothelial cells were investigated using a wound healing (scratch) assay. No statistically significant differences in wound closure rate were observed between the L-PRP-1, L-PRP-2, and P-PRP groups. However, activated L-PRP-1, L-PRP-2, and P-PRP stimulated significantly higher EA.hy926 cell motility compared to their corresponding non-activated types (Figure 2). Complete wound closure was achieved within 24 h in the presence of all activated platelet concentrates, including PFPC (Figure 2a,b). In contrast, with non-activated PRP types, wound closure was completed only after 48 h (Figure 2a,c). In the control cells (without platelet concentrates), the wound remained incompletely closed even after 48 h (Figure 2a).

Quantitative analysis of wound confluence revealed that after 24 h, the migration rate in the presence of activated L-PRP-1 was 4.7 ± 0.6-fold higher than that of non-activated L-PRP-1 (Figure 2b). For L-PRP-2 and P-PRP (prepared via two centrifugation steps), activation accelerated migration approximately 2-fold compared to the corresponding non-activated samples.

### 3.4. Effects of Activated Platelet Concentrates on Cell Proliferation

**Figure 3:**
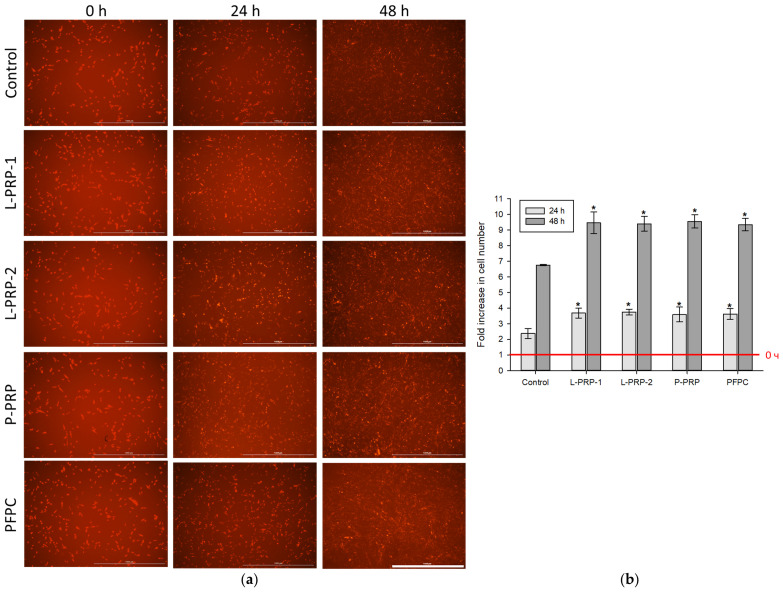
Proliferative activity of ADSCs after incubation with different types of activated platelet concentrates: (**a**) Representative fluorescence microscopy images of ADSCs labeled with PKH26. Scale bar: 1000 µm. (**b**) Quantification of viable cells determined by direct counting. * *p* < 0.05 vs. control.

Based on the results of our experiments, where it was demonstrated that pre-activation of plasma-based platelet concentrates is required for the initiation of angiogenesis and the acceleration of endothelial cell migration, we next investigated the effects of different types of activated platelet concentrates on the proliferative activity of ADSCs. A marked increase in cell number was observed across all experimental groups, including the control, indicating active proliferation of ADSCs (Figure 3). Notably, at both day 1 and day 2 of the experiment, cell monolayer density was significantly higher in all platelet concentrate-treated groups compared to the control (without platelet concentrates).

## 4. Discussion

The data obtained in this study clearly demonstrate that the methods of autologous blood fractionation and subsequent activation directly influence the functional activity of platelet concentrates. The biological evaluation was performed using ADSCs and the immortalized human endothelial cell line EA.hy926. The EA.hy926 line, generated by hybridizing primary HUVECs with A549 lung carcinoma cells, retains key endothelial characteristics, including the expression of VE-cadherin, CD31, ICAM-1, VCAM-1, and the presence of Weibel–Palade bodies [12,13]. Although these cell lines provide a robust platform for functional screening under uniform conditions, they may not fully recapitulate the complex physiological responses of primary human oral tissues. Nevertheless, the EA.hy926 line is a highly reproducible and well-validated screening model for angiogenesis research, minimizing the inter-donor variability inherent to primary cultures.

In the first phase of the work, it was established that all evaluated fractionation techniques ensure effective platelet concentration in the final product. According to the literature, a 3- to 7-fold increase in platelet concentration compared to whole blood is considered optimal for the use of platelet concentrates in regenerative medicine [14,15]. Thus, all samples obtained in our study (L-PRP, P-PRP, and PFPC) fully comply with this therapeutic range. It is well known that higher platelet concentrations are typically achieved using high centrifugation speeds and low temperatures; however, these conditions can induce premature platelet activation during centrifugation, thereby altering the regenerative properties of the platelet concentrate [16]. Consequently, the in vitro evaluation of the functional activity of our obtained fractions is of critical importance, as the most effective platelet concentration and a standardized preparation protocol for accelerating clinical wound healing remain subjects of ongoing debate. It should be noted that the experimental design relied on equivalent volumetric doses of the concentrates rather than platelet-normalized dosing across the culture well groups. While normalizing the samples by platelet count or total protein content would be required for an isolated quantitative evaluation of individual platelet efficacy, such dilution protocols would inevitably disrupt the native concentrations of plasma proteins, fibrinogen, and coagulation kinetics. Therefore, a volumetric approach was deliberately selected to preserve the authentic biochemical composition and matrix-forming capacity of each biomaterial, maintaining translational relevance to actual chairside clinical application where concentrates are delivered in their unadjusted, native volume.

Comparative analysis of the pro-angiogenic potential revealed fundamental differences between plasma-based and plasma-free forms. The necessity of pre-activating plasma-based concentrates (L-PRP and P-PRP) to trigger angiogenesis is explained by the fact that the administration of calcium and thrombin initiates platelet degranulation and a simultaneous burst release of accumulated growth factors into the medium. The robust pro-angiogenic effects observed in these activated forms corroborate data showing that L-PRP and P-PRP stimulate tubular network formation when added to EA.hy926 cells. Notably, P-PRP exerted a significantly more pronounced pro-angiogenic effect compared to L-PRP, despite similar concentrations of platelets and growth factors [17]. This discrepancy is likely explained by the high concentration of pro-inflammatory cytokines (such as IL-1β and TNF-α) inherently present in L-PRP due to the persistent leukocyte fraction. From a clinical decision-making standpoint, the presence of leukocytes in L-PRP creates a highly complex, dual-action microenvironment. While leukocytes provide crucial antibacterial defense and initiate the early phases of tissue remodeling, their subsequent release of IL-1β and TNF-α can establish a prolonged pro-inflammatory state. In endothelial cells, these cytokines activate the NF-κB signaling pathway, which can partially block or downregulate the expression of angiogenic receptors, thereby neutralizing the beneficial effects of co-released growth factors [17]. This pro-inflammatory burden is particularly critical when treating compromised tissues—such as ischemic ulcers, chronic periodontitis, or tissues affected by diabetes mellitus—where a pre-existing, non-resolving inflammatory microenvironment already dominates. In these compromised clinical scenarios, introducing an additional source of acute inflammatory mediators via L-PRP may exacerbate tissue degradation and delay defect closure. Consequently, our findings support the clinical selection of leukocyte-poor or plasma-free formulations (such as P-PRP or PFPC) for applications involving compromised tissue beds, as they minimize the localized inflammatory response while preserving potent regenerative stimuli.

A fundamentally different dynamic was observed for the PFPC in our study, which induced early vascular network formation. Such rapid angiogenesis may be attributed to the PFPC preparation technology; according to the literature, washing platelets to remove plasma after two-stage centrifugation significantly increases the content of growth factors, including VEGF, in the final platelet lysate compared to PRP [18]. An important finding was the capability of PFPC for spontaneous activation and clot formation directly within the culture well. It is worth noting that PFPC offers critical advantages over traditional PRP preparations; due to the complete removal of plasma and its constituent proteins, including fibrin and coagulation factors, various growth factors can be released in a highly bioavailable form and in larger quantities. Conversely, in native plasma-based PRP, a significant portion of regulatory molecules remains mechanically or chemically bound to the plasma fibrin matrix, which delays their diffusion to target cells. This explains why PFPC provides faster and more efficient induction of angiogenesis, making it a highly promising material for the early vascularization of bone grafts in regenerative dentistry.

It is important to acknowledge that the present study did not include direct quantitative measurement of specific growth factors (such as VEGF, PDGF, or TGF-β) in the evaluated supernatants. However, the functional cellular assays employed herein provide an integrated phenotypic readout of how target cells respond to these complex biomaterials, which arguably offers a more physiologically relevant representation of net biological activity than isolated molecular measurements. While further quantitative protein analysis could complement these findings by precisely mapping release kinetics, the observed functional outcomes already furnish robust evidence of the distinct biological efficacy of each concentrate formulation.

The stimulating effect of platelet concentrates on the directed migration of endothelial cells identified in our study fully aligns with the established literature. For instance, using time-lapse videomicroscopy in a scratch assay, Kawase et al. demonstrated that PRP effectively promotes HUVEC migration through a mechanism directly dependent on VEGFR2 activation [19]. Furthermore, the literature emphasizes the high stability of the chemotactic potential of these concentrates; even after cryopreservation, they retain the capacity to stimulate fibroblast proliferation and migration, promoting their recruitment to damaged tissue sites with an efficacy comparable to that of fresh PRP [20].

Of particular interest is the difference in the increase in migratory activity between single- and double-centrifuged forms after their activation, as recorded in our study. This phenomenon can be explained by the fact that partial mechanical activation and degranulation of platelets occur during the initial double-centrifugation processing phase [18]. Consequently, the pool of growth factors within the granules is depleted even before the activation inducer is introduced. As a result, this reduces the relative proportion of regulatory molecules released upon targeted stimulation with thrombin and calcium chloride. This substantiates a clinically important conclusion: gentle single-stage centrifugation protocols preserve the maximum intracellular potential of platelets chairside until their targeted delivery into the surgical wound, where an immediate and potent stimulus for cell migration and defect closure is required.

In the final stage of this study, based on our observation that pre-activation is required for the initiation of early angiogenesis and chemotaxis, the proliferative potential toward ADSCs was assessed exclusively using activated forms of the concentrates. The enhancing effect of platelet concentrates on ADSC proliferation is fully consistent with current data reported in the literature. It has been established that PRP significantly improves the biological properties of mesenchymal stem cells, accompanied by increased expression of genes associated with proliferation, adhesion, and cell migration [21]. Moreover, platelet lysates have been shown to support the proliferative activity of ADSCs at a level comparable to that of culture medium supplemented with fetal bovine serum [22].

The distinct kinetics and functional mechanisms identified among these fractions in vitro provide a preliminary framework for standardizing clinical protocols in regenerative dentistry and maxillofacial surgery. The rapid burst release of growth factors from activated plasma-based PRP is biologically justified for procedures requiring accelerated soft tissue healing, such as rapid epithelialization of extracted tooth sockets, socket preservation, and pain reduction—particularly in compromised patient cohorts (e.g., those with diabetes mellitus) [23]. Conversely, the ability of PFPC to induce early angiogenesis and sustainably support ADSC proliferation without inducing peak cytotoxic loads introduces promising therapeutic opportunities. In guided bone regeneration (GBR) and sinus lift procedures, where graft incorporation takes months, sustained, long-term angiogenesis is hypothesized to be critical for deep vessel ingrowth and prevention of graft resorption. Based on our 48-h in vitro findings, we propose a working model in which the rapid bioavailability of growth factors from plasma-free concentrates serves as an initial stimulus for subsequent tissue remodeling. However, because a short-term cellular assay cannot simulate complex physiological processes occurring over weeks or months, this hypothesis remains a working model that strictly requires thorough verification in appropriate long-term animal studies before any translational conclusions can be drawn regarding osseointegration quality. While these in vitro findings suggest that PFPC could serve as a potential biomimetic matrix-carrier for maxillofacial tissue engineering to optimize long-term osseointegration and enhance bone-to-implant contact, extensive in vivo and clinical studies remain strictly necessary to validate these translational claims.

## 5. Conclusions

This study is the first to perform a direct functional comparison of four types of platelet concentrates within a single experimental model to evaluate key regenerative processes. This enables the determination of optimal application niches for different platelet concentrate types, establishing a fundamental foundation for the standardization of their clinical protocols.

Single-centrifugation protocols (L-PRP-1) preserve the maximum regenerative potential of platelets within their granules until targeted activation, providing a peak (nearly 5-fold) increase in the cell migration rate. Pre-treatment with a calcium/thrombin solution for the chemical activation of plasma-based concentrates (L-PRP, P-PRP) is critically required to initiate angiogenesis and accelerate endothelial cell chemotaxis. These findings support the potential clinical utility of activated plasma-based PRP for accelerating maxillofacial soft tissue healing and preserving extracted tooth sockets, which may be particularly beneficial for pain reduction in compromised patient cohorts.

The PFPC exhibits the capacity to spontaneously form a stable clot without exogenous inducers, indicating autonomous platelet activation that leads to the early initiation of angiogenesis and efficient maintenance of ADSC proliferation in vitro. While the complete removal of plasma proteins ensures maximum bioavailability of growth factors, suggesting its potential utility as a biomimetic matrix-carrier for maxillofacial tissue engineering, extensive in vivo and clinical studies remain strictly necessary to validate its efficacy for long-term jawbone defect regeneration and sinus lift procedures.

## Figures and Tables

**Table 1:** Protocols for the preparation of different platelet concentrates.

Type	Brief Description	Key Procedural Steps
L-PRP-1	Leukocyte-Platelet-Rich Plasma (one spin)	Single centrifugation (1100 rpm, 10 min, RT) to separate erythrocytes. The supernatant containing platelets and leukocytes is collected.
L-PRP-2	Leukocyte-Platelet-Rich Plasma (two spin)	Two-step centrifugation: 1st (1100 rpm, 10 min, RT) to separate erythrocytes; supernatant collected. 2nd (3600 rpm, 15 min, RT) to pellet platelets, most supernatant (platelet-poor plasma) is removed, and the pellet is resuspended in the remaining supernatant.
P-PRP	Pure Platelet-Rich Plasma (two spin)	Two-step centrifugation: 1st (1100 rpm, 10 min, RT) to separate erythrocytes; supernatant is collected without disturbing the leukocyte layer. 2nd (3600 rpm, 15 min, RT); after centrifugation, most supernatant is removed and the pellet is resuspended as for L-PRP-2.
PFPC	Plasma-Free Platelet Concentrate	Two-step centrifugation: 1st (1100 rpm, 10 min, RT) to separate erythrocytes; supernatant collected. 2nd (3600 rpm, 15 min, RT) to pellet platelets. Key difference: After the second spin, all supernatant is discarded, and the platelet pellet is resuspended in PBS.

**Table 2:** Platelet concentration in different platelet concentrates.

Type	Initial Platelet Concentration, ×10^3^/μL	Platelet Concentration After 1st Centrifugation, ×10^3^/μL	Platelet Concentration After 2nd Centrifugation, ×10^3^/μL	Fold Increase in Platelet Concentration
L-PRP-1	221.0 ± 49.3	779.7 ± 312.4	-	3.42 ± 0.77
L-PRP-2	315.0 ± 66.2	986.7 ± 404.6	2063.3 ± 830.8	6.36 ± 1.34
P-PRP	241.0 ± 57.2	653.3 ± 295.2	1948.3 ± 657.9	7.97 ± 0.86
PFPC	205.7 ± 41.7	681.7 ± 266.2	1826.3 ± 921.4	8.62 ± 2.76

**Table 3:** Leukocyte concentration in different platelet concentrates.

Type	Initial Leukocyte Concentration, ×10^3^/μL	Leukocyte Concentration After 1st Centrifugation, ×10^3^/μL	Leukocyte Concentration After 2nd Centrifugation, ×10^3^/μL	Fold Decrease in Leukocyte Concentration
L-PRP-1	4.9 ± 1.1	1.1 ± 0.5	-	4.45 ± 2.20
L-PRP-2	6.1 ± 0.8	1.0 ± 0.8	1.5 ± 1.0	3.96 ± 0.79
P-PRP	7.3 ± 1.5	0.6 ± 0.6	0.7 ± 0.6	10.43 ± 3.85
PFPC	9.8 ± 1.1	2.6 ± 0.7	3.9 ± 1.5	2.49 ± 0.74

## Data Availability

The original contributions presented in the study are included in the article; further inquiries can be directed to the corresponding author.

## Abbreviations

ADSCs	Adipose Derived Stem Cells
HUVECs	Human umbilical vein endothelial cells
L-PRP-1	Leukocyte-Platelet-Rich Plasma, one spin
L-PRP-2	Leukocyte-Platelet-Rich Plasma, two spin
PFPC	Plasma-Free Platelet Concentrate
PRP	Platelet-Rich Plasma
P-PRP	Pure Platelet-Rich Plasma
